# Crucial Role Reported for TSPO in Viability and Steroidogenesis is a Misconception. Commentary: Conditional Steroidogenic Cell-Targeted Deletion of TSPO Unveils a Crucial Role in Viability and Hormone-Dependent Steroid Formation

**DOI:** 10.3389/fendo.2016.00091

**Published:** 2016-07-18

**Authors:** Vimal Selvaraj, Lan N. Tu, Douglas M. Stocco

**Affiliations:** ^1^Department of Animal Science, Cornell University, Ithaca, NY, USA; ^2^Department of Cell Biology and Biochemistry, Texas Tech University Health Sciences Center, Lubbock, TX, USA

**Keywords:** translocator protein TSPO, steroid biosynthesis, mitochondria, adrenal cortex, Leydig cells, cholesterol, embryonic lethality, lipid metabolism

Recent reports on Leydig cell-specific *Tspo* conditional knockout *Tspo^c^*^Δ/Δ^ mice (1), viable global *Tspo* knockout (*Tspo^−/−^*) mice from two independent laboratories (2, 3), and clones of CRISPR/Cas9-mediated *Tspo*-deleted MA-10 Leydig cells (MA-10*^Tspo^*^Δ/Δ^) (4) established that TSPO is not essential for steroid hormone biosynthesis or viability [reviewed in Ref. (5, 6)]. These reports refuted 25 years of dogma that described TSPO as a mitochondrial cholesterol transport protein, indispensable for steroidogenesis. In response, the research group involved in most of the early studies linking TSPO and steroidogenesis investigated Leydig cell-specific and adrenocortical cell-specific *Tspo^c^*^Δ/Δ^ mice (7) and presented results that seem to repudiate the recent findings and revive the old model. In this commentary, we would like to point out that interpretations made in the manuscript by Fan et al. (7) are seriously flawed.

## TSPO Deletion Does Not Affect Viability

**Figure 1:**
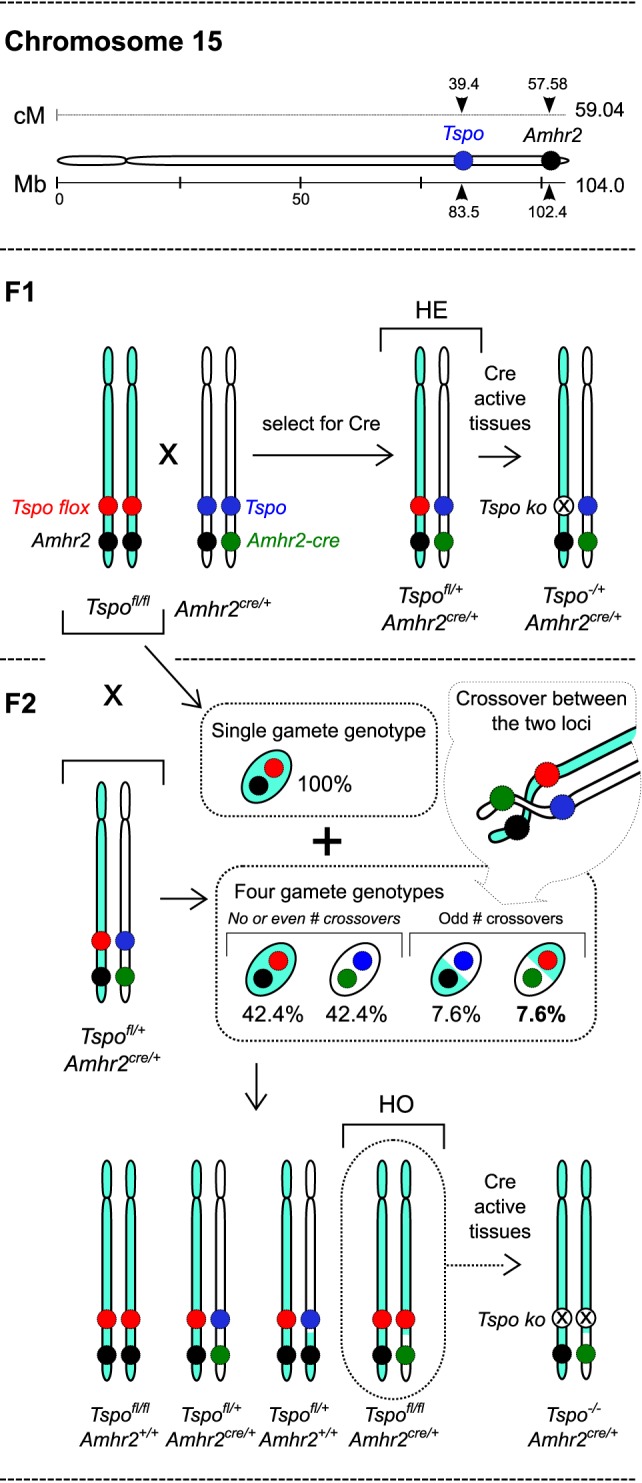
**“Embryonic lethality” based on a miscalculation: inheritance of the *Tspo* and *Amhr2* gene loci in chromosome 15 with respective floxed and knock-in cre alleles**. Murine chromosome 15 showing the physical positions (in megabases – Mb) and genetic positions (in centimorgans – cM) of *Tspo* and *Amhr2* genes (drawn to scale). F1: breeding between *Tspo^fl/fl^Amhr2*^+/+^ and *Tspo*^+/+^*Amhr2^Cre/^*^+^ can produce cre positive heterozygous *Tspo*^fl/+^*Amhr2*^Cre/+^ (HE) offspring and *Tspo*^fl/+^*Amhr2*^+/+^ offspring (not shown). With tissue-specific cre drivers, there is recombination in *cre* expressing cells leading to *Tspo^−^*^/+^*Amhr2*^Cre/+^ HE mono-allelic knockout cells. F2: HE mice are backcrossed with *Tspo^fl/fl^Amhr2*^+/+^ mice in order to generate *Tspo*^fl/fl^, *Amhr2*^Cre/+^ (HO) mice. The *Tspo^fl/fl^Amhr2*^+/+^ mice produce only one gamete genotype, whereas HE mice produce four gamete genotypes, the ratio of which depends on the frequency of odd number of crossovers that occur between the *Tspo* and *Amhr2* loci, and not through independent assortment, because they are in the same chromosome. Consequently, the gamete genotype necessary for generating HO offspring occurs at a calculated frequency of 7.6%. This is much lower than the classic Mendelian ratio of 25% because *Tspo* and *Amhr2* gene loci are closely spaced in chromosome 15, and are, therefore, linked and inherited together at a high frequency. The experimental value of 4.4% observed by Fan et al. (7), is anticipated and indicates the biological value for that particular mouse strain. Therefore, interpretation of embryonic lethality in HO mice based on an incorrect expectation of 25% by Fan et al. (7) is seriously flawed.

In Fan et al. (7), it was observed that use of *Amhr2^cre/^*^+^ knock-in mice (8) to generate Leydig cell-specific *Tspo^c^*^Δ/Δ^ mice resulted in low Mendelian ratios for homozygous cre positive mice (HO: *Amhr2^cre/^*^+^*Tspo^c^*^Δ/Δ^). This was interpreted as partial preimplantation embryo loss, and the authors concluded that TSPO is crucial for viability. This is a fundamental mistake because both *Amhr2* and *Tspo* are in the same chromosome 15 and, therefore, cannot assort independently. The *Tspo* and *Amhr2* genetic positions are just 18.18 cM apart, and the probability for chromosomal crossover between the two loci to get HO mice is calculated as 7.6% [based on Haldane (9), other estimates are similar]. Therefore, the low rate of 4.4% HO mice observed by Fan et al. (7) is anticipated and represents the precise biological value due to linkage of the two loci and is certainly not an indication of embryonic lethality (Figure 1) [Note: Actual values are not identical to calculated numbers because of differences in mouse strain, secondary regulation, specific chromosome structures, and interference at crossover sites]. Expectation of 25% HO mice based on classical Mendelian principles is not applicable in this context, and the interpretation made on this basis is highly inaccurate.

To explain their proposed case of embryonic lethality, Fan et al. (7) proposed that the *Amhr2^cre/^*^+^ knock-in mice used to generate gonadal cell type-specific conditional deletions in more than 90 publications (MGI ID: 3042214), in their particular case, could induce global *Tspo* deletions. The justification provided was published microarray datasets that seemed to detect (with inconsistencies) an increase in *Amhr2* transcription in 2-cell embryos. There was no primary data in the manuscript validating this assertion that appears highly unlikely. Even if it were to occur, global *Tspo* deletions would not affect the linkage and rate of HO mice as described above. In previous studies that used cre on different chromosomes, Mendelian ratios were observed during generation of viable global *Tspo^−/−^* mice with similar mouse backgrounds (2, 3).

## TSPO is Not Involved in Steroidogenesis

In Fan et al. (7), the *Nr5a1^cre^Tspo^c^*^Δ/Δ^ mice showed expected *Tspo* deletions in Leydig cells and the adrenal cortex. Testosterone production (both baseline and after induction using hCG) was not affected, consistent with the previous report (1). Although the authors note this as “surprising,” the significance of this observation as indication that TSPO was not involved in mitochondrial cholesterol import in Leydig cells was disregarded. It is not our intention to criticize, but we are indeed under obligation to point out that the proclaimed landmark *in vitro* studies of *Tspo* disruption (10) and *Tspo* knockdown (11) used as foundations for asserting TSPO link to steroidogenesis were performed only using Leydig cells by this same research group. These *in vitro* results have since not been reproducible both *in vitro* (2, 4) and *in vivo* (1, 2) and is now also invalidated by Fan et al. (7) without an explanation.

In *Nr5a1^cre^Tspo^c^*^Δ/Δ^ mice, baseline corticosterone levels were also unaffected, consistent with the previous report (2). However, when induced with ACTH, circulating corticosterone did not increase in both heterozygous (*Tspo^c^*^Δ/+^) and homozygous (*Tspo^c^*^Δ/Δ^) deletions of *Tspo* compared to *Tspo^fl/fl^* controls, an observation that had no correlation to TSPO expression levels and was in contrast to the previous report (2). This inconsistency and differences observed with regard to lipid accumulation and changes to *Lhcgr* and *Scarb1* expression in *Nr5a1^cre^Tspo^c^*^Δ/Δ^ adrenals (7) may be linked to parallel findings showing that TSPO can affect lipid metabolism in cells (12). Although additional work is necessary, by attempting to provide explanations based on the unsubstantiated conjecture that TSPO is an essential cholesterol transport protein for steroidogenesis, the authors have missed an excellent opportunity to advance understanding of TSPO function.

## Concluding Remarks

Global *Tspo^−/−^* mice are viable (2, 3) and are an excellent tool for investigating TSPO function in health and disease. Evidence refuting TSPO link to steroidogenesis is not based only on reports in *Tspo^−/−^* mice (2, 3). Recent studies, using *Tspo*-deficient isolated mitochondria (3), *in vitro Tspo* knockdown (2), and CRISPR/Cas9-mediated *Tspo* knockout in cell lines (4), have all highlighted problems with reproducibility of prior work. The serious misinterpretations made in Fan et al. (7) are negatively impacting research progress across multiple fields and distracting from pursuit of the core function of TSPO.

## Author Contributions

All authors listed have made substantial, direct, and intellectual contribution to the work and approved it for publication.

## Conflict of Interest Statement

The authors declare that the research was conducted in the absence of any commercial or financial relationships that could be construed as a potential conflict of interest.
